# CONCEPT ANALYSIS: SLEEP QUALITY IN OLDER ADULTS

**DOI:** 10.1093/geroni/igae098.3517

**Published:** 2024-12-31

**Authors:** Suah Park, Eeeseung Byun

**Affiliations:** University of Washington, Seattle, Washington, United States; University of Washington School of Nursing, Seattle, Washington, United States

## Abstract

Sleep quality is crucial in determining one’s overall quality of life, particularly among older adults disproportionately affected by poor sleep. Despite its significance, there exists a gap in the analysis of the concept of sleep quality in older adults. This concept analysis was guided by Walker and Avant’s method to conceptualize sleep quality in older adults comprehensively. A systematic search of studies published between 2018 and 2023 was conducted across databases, including CINAHL, EMBASE, PubMed, and Web of Science. Inclusion criteria encompassed quantitative and qualitative studies exploring sleep quality in older adults. The analysis identified key elements, such as defining attributes, antecedents, and consequences of sleep quality in older adults, based on the content of the articles. Fifty articles provided data for sleep quality in older adult conceptualization. Six defining attributes were identified: (a) number of awakenings, (b) sleep duration, (c) sleep disturbance, (d) sleep efficiency, (e) sleep latency, and (f) sleep satisfaction. Antecedents encompassed factors such as Diet, Physical activity, Circadian rhythm, Perceived health, Chronic disease, Psychosocial factors, Sociodemographic factors, and Environmental factors. Consequences included Fall, Daytime dysfunction, Physical health, Mental health, Mortality, and Quality of life. The bidirectional relationships between consequences and sleep quality indicated that some antecedents could be part of consequences and vice versa. This concept analysis presents a conceptual model of sleep quality in older adults based on defining attributes, antecedents, and consequences. The model provides a foundation for further testing and developing measures and interventions for addressing poor sleep quality in older adults.

